# Creating a best practice template for participant communication plans in global health clinical studies

**DOI:** 10.1186/s13063-023-07185-4

**Published:** 2023-03-02

**Authors:** Colleen E. Shelly, Caroline Logan, Beth Skorochod, Alison Wiyeh, Duduzile Ndwandwe, Augustine Choko, Innocent Valea, Boghuma K. Titanji

**Affiliations:** 1 HealthAnalytix, LLC, 85 Larch St., Providence, RI USA; 2 CollaborateUp Inc, 208 Noland Street, Falls Church, VA USA; 3 Department of Epidemiology, School of Public Health, University of Washington, Seattle, WA USA; 4 Cochrane South Africa, South African Medical Research Council, Francie Van Zilj Drive, Parow Valley, Cape Town, South Africa; 5 The Clinical Research Unit of Nanoro (IRSS-CRUN), Nanoro, Burkina Faso; 6 Malawi Liverpool Wellcome Trust Clinical Research Programme, Queen Elizabeth Central Hospital College of Medicine, Blantyre 3, Malawi, C, P.O. Box 30096, Chichiri, South Africa; 7 Emory University School of Medicine, 2015 Uppergate Dr, Atlanta, GA USA

**Keywords:** Communication plan, Clinical trial, Global health, Clinical trial participant

## Abstract

**Background:**

Clinical trial participants have a right to be informed throughout the entire process of human subject research. As part of this pillar of research ethics, participants and other stakeholders should be made aware of research findings after a trial has been completed. Though participants have both a right, and a desire to be informed of research outcomes, studies show that they rarely receive communication about study findings. Our aim was (1) to understand what, if any, role communication plans play in current global health clinical research protocols and (2) to use our findings to develop a communication plan template tailored to clinical research carried out in low-and-middle-income countries (LMIC) while minimizing colonial assumptions. While the template was drafted in the LMIC context, the principles are universally applicable and should be considered best practices for all global health clinical trials.

**Methods:**

We carried out a mixed-method study over a period of 6 months to understand the role of communication with study participants and other stakeholders in clinical trials. The semiquantitative analysis included mining publicly available clinical trial protocols for communication-related language. Qualitative interviews (*n* = 7) were used to gather knowledge and insight from clinical trial experts to inform the development of a communication plan template.

**Results:**

None of the 48 mined clinical trial protocols included a communication plan. Of the 48, 21% (*n* = 21) protocols included communication-related language, and 10% (*n* = 5) described plans to share trial results with participants.

**Conclusion:**

The use of communication plans in global health clinical trials is lacking. To our knowledge, this is the first in-depth analysis of communication plans in clinical trials to date. We recommend that researchers utilize the developed communication plan template throughout the entire research process to ensure a human-centered approach to participant communication. This communication plan should apply to all phases of a research trial, with a particular emphasis on plans to share results in an accessible and engaging manner once the trial has been completed.

**Supplementary Information:**

The online version contains supplementary material available at 10.1186/s13063-023-07185-4.

## Introduction

Respect for persons is the first basic ethical principle when carrying out human subject research [1]. Within this pillar of clinical research ethics, outlined by the Declaration of Helsinki, is an obligation to inform participants throughout the entire research process, including after a clinical trial ends [2]. It is critical that the information is communicated to study participants effectively, to avoid distress or confusion [3]. Effective communication and sharing of research results with participants and other stakeholders is vital to the success of a clinical trial, as it can improve recruitment and retention, build trust through transparency, foster collaboration, and reduce health disparities [1, 4]. Moreover, research participants may feel more valued when the channels of communication remain intact before, during, and after their participation.

Increasingly, researchers are disseminating the findings of their studies through presentations at academic conferences and scientific publications. These means of dissemination tend to be better suited for other fellow researchers and less so for trial participants, who are often people from the lay public [3]. Although most research participants want to be made aware of research findings [5–9], few ever receive information on clinical trial findings in practice [9, 10]. One survey reported that only a third of respondents who had previously volunteered in research reported having received research results [9]. Several factors may contribute to the lack of dissemination, including limited interest of participants, a mismatch between the selected communication style and the audience, challenges with reaching participants after a trial is completed, a lack of standardization and early prioritization of dissemination activities, sharing only “interesting” findings, or concerns over how to share “negative” results [1, 10, 11].

There are several ongoing efforts aimed at improving the dissemination of research findings to key stakeholders. The National Health Institute’s 2003 Data Sharing Policy requires the inclusion of a data sharing plan in research proposals over $500,000 in direct costs [12]. The Food and Drug Administration Amendments Act (FDAAA) of 2007 aimed to build on previous data sharing regulations and highlighted a requirement for the reporting of adverse events and summary results for FDA-approved products [13]. Nine years later, the Final Rule was implemented to improve effectiveness and increase compliance with reporting guidelines set forth by the FDAAA [14, 15]. The Final Rule also required the inclusion of a full protocol and a statistical analysis plan (SAP) [14, 16].

Along with the Final Rule, the 2016 National Institute of Health (NIH) Policy on the Dissemination of NIH-Funded Clinical Trial Information requires investigators with partial or full NIH funding to register and report summarized results of interventional clinical trials to ClinicalTrials.gov [12]. Similarly, The World Health Organization (WHO) passed a resolution in May 2022 outlining its support for measures to “facilitate the timely reporting of both positive and negative interpretable clinical trial results,” through the registration of results and publication of findings [17]. Though these regulations intend to increase reporting and public access to information, a recent study found that roughly 40% of registered trials reported results within the 1-year deadline, and only 63.8% reported results at any time. This cohort study observed no improvement in compliance from 2018 to 2019 [15]. The WHO reported in 2017 that less than half of clinical trials publicly report results, often due to null or negative findings [18]. Unlike requirements to ensure the reporting of data sharing plans, registration of trials, and publication of results, there are currently no requirements with regard to communication between investigators, study participants, and their communities [12]. Standardizing the participant communication process could be helpful in increasing compliance.

When developing a communication strategy, researchers should also consider how participants and communities may share the benefits of clinical research activities by asking members of the community what benefits they would find most impactful. Benefit sharing is part of a broader concept of increasing transparency and building trust in the research process. Providing a framework for benefit sharing as a required component of a communication plan serves as a tangible action to improve equity and build trust in the broader research process [19].

The aim of this study was to use qualitative and quantitative methods to (1) understand the current climate of communication language and communication plans in global health clinical trials in low-and-middle-income countries (LMICs) and (2) develop a communication plan template for clinical researchers to incorporate into their protocols, outlining communication strategies with all priority stakeholders throughout the entirety of a trial and defining methods of sharing clinical trial results once a trial has ended.

## Methods

**Fig. 1:**
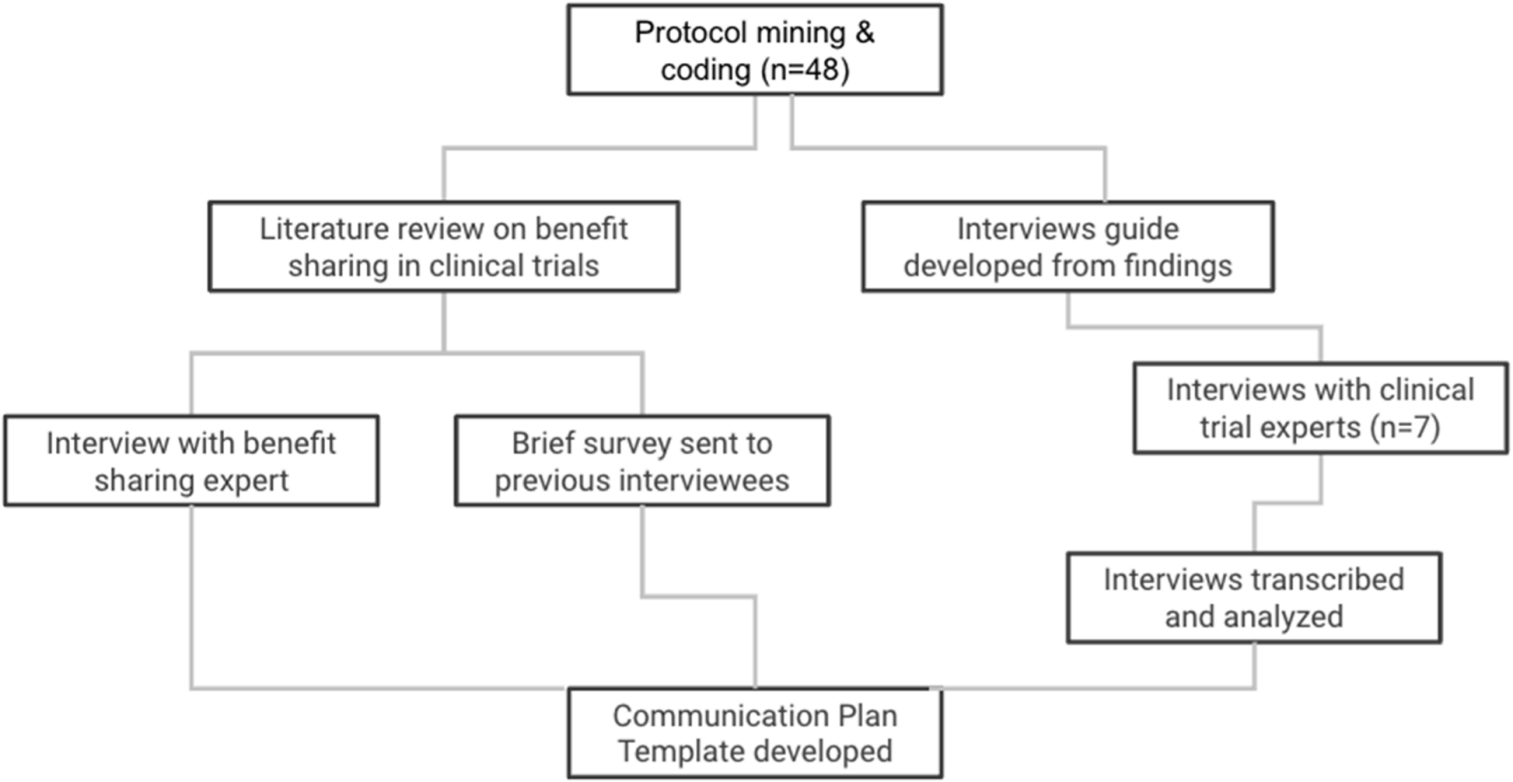
Study schema

We carried out a three-pronged approach, consisting of (1) an assessment of recent clinical trial protocols for the incorporation of communication plans, (2) in-depth interviews with subject matter experts, and (3) a literature review of existing communication plans (Fig. 1).

### Protocol mining

We mined protocols sourced from the DAC Protocol Library on The Global Health Network [19] for language related to communication and communication plans. The DAC Protocol Library was utilized as a known resource already enriched with the types of protocols most relevant to the search criteria. Included protocols were full protocols published between 2015 and 2019 that had to describe a study carried out in an LMIC in the following disease areas: human immunodeficiency virus (HIV), neglected tropical diseases, tuberculosis (TB), malaria, maternal, newborn, and child health, nutrition, vaccines, enteric and diarrheal diseases, pneumonia, and polio. Protocols of study designs other than randomized control trials were excluded. Protocols were reviewed and consistently coded (yes/no) for the inclusion of any communication-related language (i.e., anything relating to communication with clinical trial participants, communities, or other stakeholders). Any information related to communication, regardless of relevance to communication plans, was objectively abstracted and further categorized into the following groups based on codes agreed upon by the study team: communication language (general), communication plan, community engagement, community advisory board, publicly available, results shared with local authorities, and results shared with participants. Data were recorded in Microsoft Excel. Descriptive statistics on the existence of, type, and frequency of communication language were carried out.

### Qualitative interviews

In-depth interviews were conducted with a variety of clinical trial experts (principal investigators, nurses, clinical trial managers, etc.) from a wide array of organizations who each have experience conducting clinical trials in LMICs. An interview guide was developed based on protocol mining and desk research, with questions carefully crafted to understand (1) participants’ experiences with effective and ineffective communication practices pre-trial, during the trial, and post-trial and (2) participants’ suggestions and best practices for impactful strategies to improve communication with clinical trial participants and the broader community (see Additional file 1). Potential interview participants were chosen as a result of their extensive experience designing and executing multiple clinical trials in LMIC countries. During interviews with these identified individuals, several offered names of additional experts to consult given the nature of their experience and research. As a result of these recommendations, additional experts were interviewed. An introductory email was sent out to each potential participant with a brief description of the research and context surrounding the process for the creation of a useful communication template for global health clinical trials. Interviews were set up through email at the respondents’ preferred time and carried out and recorded via Zoom using an interview guide. Verbal consent was obtained prior to recording each interview, and participants were reminded that they could stop the interview at any time. In addition to the recording, notes were taken by two members of the study team for each interview and verified with the transcription to ensure accuracy and maintain rigor. Participants agreed to allow all interview data to be analyzed in aggregate. As this study was a qualitative and quantitative review of publicly available protocols and interviews with subject matter experts, we did not seek approval from an institutional review board.

### Interview transcription and analysis

Interview recordings were imported into NVivo software NVivo (RRID:SCR_014802) and transcribed verbatim using the transcription function. The transcriptions were imported into Microsoft Word and content was validated by comparing the audio recordings to the transcribed output. Transcriptions were edited where necessary. Interviews were read and listened to twice for familiarization and then coded for communication content and important themes. Thematic content analysis was performed using the coded interviews and interview notes taken by members of the study team. Findings are summarized in Table 1.

**Table 1:** Themes identified through interviews with clinical trial experts

Communication plan	•Perform stakeholder mapping to identify those who influence decision-making and those impacted by the decisions •Identify who, when, and how to engage •Have a monitoring and reevaluation plan to assess if the communication plan is working •Identify benchmarks of success •Describe the strategy, audience, and what needs to be done prior to implementing the plan •Include information about what will happen after the trial •Conduct meetings with stakeholders before the start of a trial to get the appropriate permissions
Barriers and challenges	•Spread of misinformation, distrust •Not setting the correct expectations •Low literacy levels •Decay in communication over the lifespan of the trial •Community exhaustion from previous trials •Not speaking to community leaders prior to trial commencement
Best practices	•Include a community advisory board (CAB) and have regular meetings to help develop communication tools and advocate for the trial •Share results of the trial with the broader community so that others see the potential benefits of participating in clinical trials •Share results with nurses and trusted clinical staff to help communicate results •Use simple and understandable messages
Channels of communication	•Phone, text/WhatsApp •Local radio •Theater •Fliers •Email
Post-trial communication	•Gather chiefs and peer leaders to share results and thank them for participating •Incorporate a referral system, if appropriate •Conduct meetings/workshops with the public •Share the scientific publication •Share findings with healthcare professionals

### Benefit sharing

A brief, four-question survey was sent to interview participants using Google Forms, with an aim to understand the participants’ knowledge of benefit sharing and any potential experience incorporating it into clinical trials. An additional literature review was carried out to gain insight on benefit sharing in the current clinical trial context. A recognized author and thought leader on the subject, with a recently published paper on benefit sharing, was identified through the review process. The research team contacted the author, and a brief interview was carried out. Notes were taken during the interview and summarized to help inform the addition of benefit sharing language in the communication plan template.

### Communication plan template

A comprehensive narrative literature review was conducted to assess additional published papers discussing communication plans in clinical trials. Citations from each reviewed paper deemed relevant were used to identify additional literature. Findings from the literature review were combined with quantitative and qualitative findings, and an initial communication plan template was developed. The first draft of the template was sent to each interviewee for feedback. A total of two reminder emails were sent, and feedback was received from five of the seven participants. The template was then circulated with members of the Bill & Melinda Gates Foundation’s DAC team for additional comments. Feedback was incorporated into a second communication template draft.

## Results

### Protocol mining

**Fig. 2:**
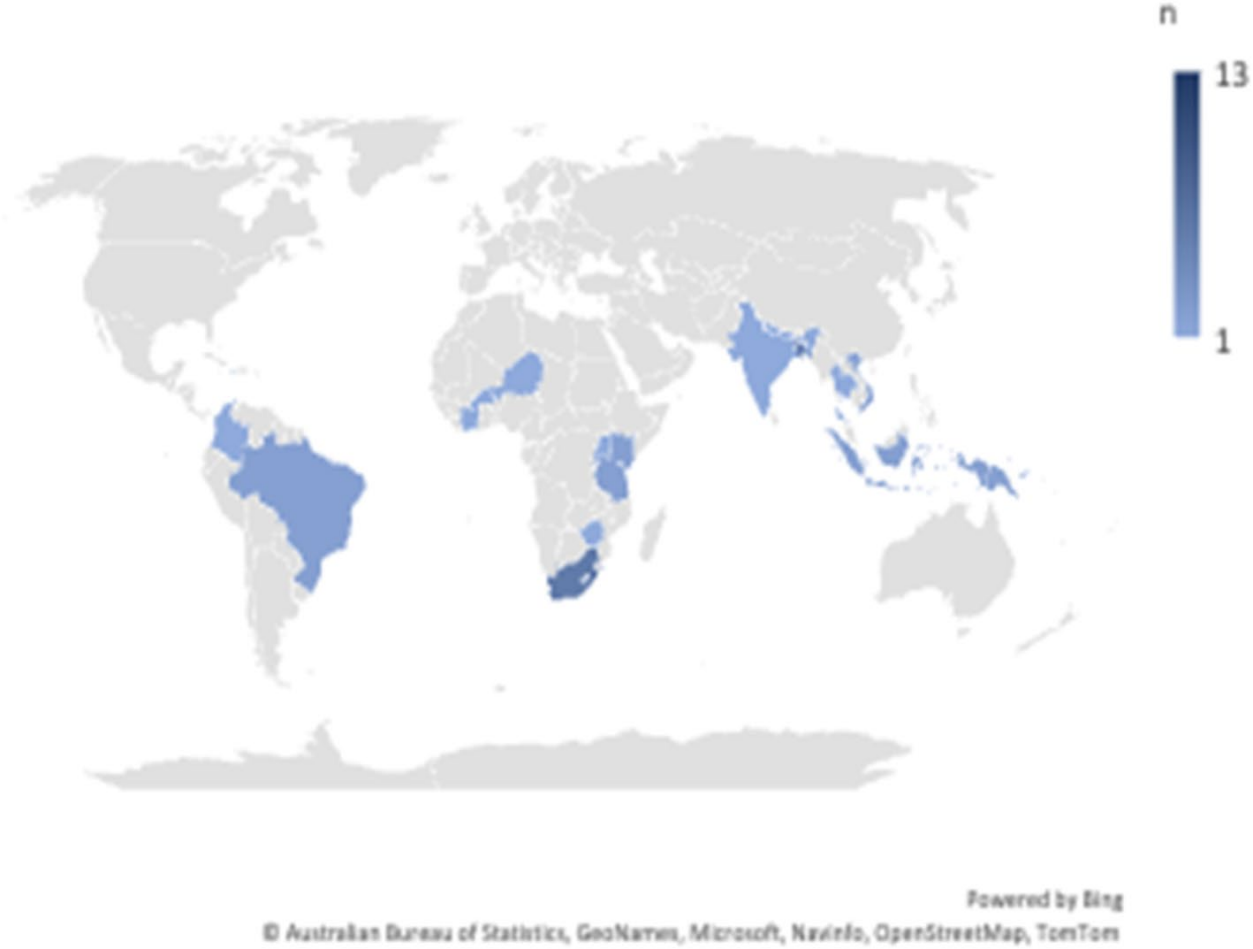
Countries where research was carried out from 48 mined protocols

**Fig. 3:**
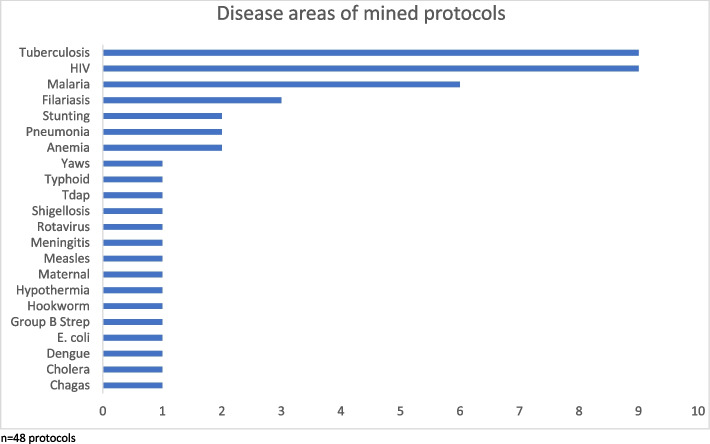
Disease areas of mined protocols

We identified and reviewed 48 full clinical trial protocols from the Global Health Network website [20]. The protocols described studies conducted in countries such as: South Africa (*n* = 6), Bangladesh (*n* = 5), Kenya (*n* = 2), and 20 other LMIC countries (data reported in Table 2 and displayed in Fig. 2). Protocols described research in areas such as HIV, Malaria, TB, Filariasis, and other (data reported in Fig. 3). The following categories were recorded from metadata of mined protocols: communication language, communication plan, community engagement, community advisory board, publicly available, results shared with local authorities, and results shared with participants. Though 44% (21/48) of protocols included communication language, only one protocol mentioned a communication plan but did not include one in the document. Ten percent (5/48) of protocols detailed plans to share trial results with participants (Table 3).

**Table 2:** Number of protocols by country from 48 mined protocols

Country	*n*
Multicountryª	13
Bangladesh	5
Brazil	2
Burkina Faso	1
Colombia	1
Haiti	1
India	1
Indonesia	2
Ivory Coast	1
Kenya	2
Lesotho	1
Nepal	1
Niger	1
Papua New Guinea	2
Rwanda	1
South Africa	6
Tanzania	2
Thailand	1
Uganda	1
Vietnam	2
Zimbabwe	1

^a^Multicountry is defined as a protocol describing a study carried out in two or more countries

**Table 3:** Codes established from protocol mining

Protocol characteristic	*n* (%)
Had communication plan	0 (0%)
Discussed a communication plan	1 (2%)
Had communication language	21 (44%)
Mentioned community engagement	3 (6%)
Mentioned community advisory board (CAB)	3 (6%)
Mentioned that results will be made publicly available	3 (6%)
Mentioned they would share results with local authorities	3 (6%)
Detailed plans to share results with participants	5 (10%)
Included information about communicating with community, including for recruitment purposes, permission to carry out study, or community feedback	5 (10%)

Number and proportion of protocols that have each protocol characteristic of all 48 mined protocols

### Qualitative interviews

Out of the eleven clinical trial experts invited to participate in the interview process, seven agreed to participate, and interviews took place between December 2021 and January 2022. Each interview lasted approximately 45 min and was recorded on Zoom. Most participants had more than a decade of experience in clinical trials, including experience as clinical trial coordinators, nurses, doctors, lab supervisors, and principal investigators. All participants had experience in clinical trials in Africa, including Kenya, Uganda, Malawi, Tanzania, Cameroon, Senegal, Burkina Faso, Nigeria, Rwanda, and South Africa. Several experts had experience conducting trials globally, including in the USA and Latin America. The following codes/categories were identified through qualitative interviews carried out with experts in clinical trials: communication plan/community engagement plan, barriers and challenges, communication channels, post-trial communication, community advisory board, milestones/benchmarks, role in clinical trials, stakeholders, and trust (Table 3).

### Benefit sharing

One participant completed the benefit sharing survey, stating that they were aware of the concept, but that it is “not really clear how benefit sharing works” within clinical trials. They commented that “community members, including stakeholders, [should] hold the research team accountable based on the benefit sharing principle… This, in turn, would [ensure] participants understand that their participation in a trial is for a greater good.”

In our literature review of benefit sharing in clinical trials, we found that benefit sharing is most often discussed in the context of human genomic information and data sharing. The concept itself was developed alongside the forming of the Nagoya Protocol, which outlines three objectives: “conservation of biological diversity, the sustainable use of its components, and the fair and equitable sharing of benefits arising from the utilization of genetic resources” [21, 22]. Bedeker et al. defined benefit sharing as “the actions taken towards ensuring that various benefits of research are shared with a wide range of stakeholders in a way that is equitable and just.” Examples in clinical trials may include sharing financial benefits with trial participants, improving infrastructure in the communities where the research takes place, or providing opportunities for career advancement. They note that although a data sharing plan is typically required when applying for research funding or disseminating findings, benefit sharing is not [19]. In an interview with Dr. Nicki Tiffin, professor at the South African National Bioinformatics Institute and author on the same paper, she emphasized the barriers to effective benefit sharing in current research programs, including a lack of tangibility, difficulties with operationalization, and limited communication or transparency about the research process. She suggested that incorporating benefit sharing into a trial or community engagement plans can be simple and uncomplicated and noted that researchers should start by asking the participants and community what would be beneficial to them. She noted, “..[a benefit] could be as simple as buying three new refrigerators for a clinic, or [improving] a waiting room at a clinic.” For example, in a community in Botswana, the Collaborative African Genomic Network (CAfGEN) implemented a community engagement project in which they distributed print comics to explain sickle cell disease. This helped introduce children to genomics and sickle cell disease in an engaging way, while launching a career for the cartoonist in the process who benefited financially and reputationally from this work [23].

Dr. Tiffin urged researchers to “speak to people in the community where participants are recruited and ask what would be useful… don’t decide for the stakeholders”. If a team has a specific budget, provide the community with a set of options. This engagement could take the form of an open community focus group to inquire as to what kind of benefits they would like to see as a part of the trial in order to avoid making assumptions about what the community wants/needs. From the options voiced and in consultation with the research team on what is feasible, the community could vote to determine the benefits they would like to see most. Dr. Tiffin suggested that another hurdle in the process of implementing benefit sharing is that the conversation often gets stuck at the researcher level, deciding who gets credited, which universities receive funding, and who gets invited to conferences with other researchers. At a macro level, the failure to consciously share benefits of scientific progress with the general public through translation into public health and other benefits has impeded public support for evidence-based, scientific responses even while benefits have accrued to meso and macro stakeholders. Providing a framework to move beyond the researcher level and into the community stakeholder level is critical to overcome these challenges and to build trust in the research process more broadly (N. Tiffin, Personal Communication, 11 May 2022).

### Communication plan template

The final version of the Communication Plan Template can be downloaded here. This communication plan template was developed to support effective, inclusive communication to all stakeholders, including clinical trial participants and their broader community, before, during, and after a clinical trial. The results from our research and interview process revealed several key findings including:

- The need for a communications plan pre-trial, during the trial, and after the trial’s conclusion.
- The need to not only prioritize communications with trial participants but to ensure an effective communication strategy for the surrounding community. While all audiences are important, there is a need to prioritize engaging key influencers in the community who can amplify positive messages and communicate benefits to the community in a clear and compelling way.
- The utility of utilizing a Community Advisory Board (CAB) with the caveat that developing a CAB will require an inclusive, deliberate, and thoughtful approach.
- The need to work with communities to understand what is important to them in order to craft impactful key messages.
- The need to understand the channels best suited to communicate with priority stakeholders; not only what they have access to, but what they prefer, depend on, and trust.

Given the learnings identified, we sought to develop a template that clearly outlines steps for effective communications pre-trial, during trial, and post-trial. Pre-trial communications steps outlined in the template include:

- Understanding priority stakeholders and identifying shared benefits: The most effective communications are targeted. Targeted communications start with identifying and understanding priority stakeholders. This process in turn allows for the grouping of stakeholders into subsets that share similar characteristics or needs for information. This section outlines how to collect information to understand your priority stakeholders’ awareness, beliefs, and feelings about the issues being researched; influencers and what is most important to them; and preferred channels of communication. This section also outlines how to gather information in order to understand what kinds of benefits priority stakeholders would like to see from the trial, allowing trial teams to hone in on what is useful for the community, without making assumptions about the kinds of benefits the community is interested in receiving.
- Priority stakeholder mapping: After a desk review and interactions with community stakeholders in the prior step, stakeholder mapping asks users to determine who will be the most important to prioritize for communications. This section of the template allows users to consider priority stakeholders, their contact information, who they communicate with, how this stakeholder group may benefit from the trial, and other important considerations.
- Develop communication objectives and key messages: This section of the template outlines the importance of working with trial organizers to develop key messages surrounding the trial. Objectives should be designed to empower trial participants and the communities that surround them to understand the trial and its intent, building trust for clinical research and the institution or health facilities implementing the clinical research.
- Crisis communication planning: While the template does not provide a comprehensive overview of crisis communication planning, it emphasizes what crisis communication planning entails, how to ensure benefit sharing is utilized for participants and the community even in times of crisis, and how to assemble a diverse communications team proactively before a crisis occurs.
- Determine communication channels: Based on the review of priority stakeholder groups in prior steps, this step allows users to identify the appropriate channels best suited to deliver messages, the mix of communication channels required, and the ideal frequency of communications. It asks users to identify the channel, note which priority stakeholder group it reaches, and indicate which phase of the trial will use this channel (pre-trial, during trial, or post-trial).
- Consent and expectation setting: This section notes the importance of participant consent and community assent. It asks users how they will assess informed consent and address risk perceptions with priority stakeholders.
- Consider a community advisory board (CAB): This section advises that researchers utilize a community advisory board (CAB). CABs act as a consistent conduit of information both from and to the community around the trial and can be instrumental in understanding the cultural norms and social values that can influence a communication plan. Establishing a CAB is labor and time intensive. Once established, it needs to be nurtured throughout the trial. The guiding questions in this section outline important considerations for CAB development, should the trial design team choose to use a CAB.

The during trial communications section of this template includes:

- Communication implementation plan: In the pre-trial communications section of the template, users are asked to identify priority stakeholders, create communication objectives, and develop key messages. Before the monitoring and evaluation phase, research teams must craft a communication implementation plan. This section of the template asks the user to outline the priority stakeholder group, key message, channel or activity, frequency, timing, and cost for each communication activity. This section also provides best practices around the use of infographics, as a sub-component of the communication implementation plan.
- Monitoring and evaluation: This section of the template outlines how users will monitor the process and the quality of communications. This section of the template includes a guide for communication indicators such as retention rates. It asks users to consider how to measure the extent to which their communications are succeeding—and whether priority stakeholders are receiving and retaining key messages. In addition, it asks users to monitor whether communities are receiving the shared benefits outlined in the pre-trial communications section and, if not, how they might adjust in order for them to do so.
- Common challenges and tactics to address them: While it is impossible to predict the kinds of challenges that will emerge in a clinical trial, this section outlines several potential communication issues and associated mitigation strategies drawn from our research and interview process. This section is not an exhaustive list but is intended to encourage users to be proactive, anticipating challenges and developing communications activities accordingly.

The post-trial communications section focuses on post-trial findings. This section outlines how to share trial results, informing clinical trial participants and the wider community. This section also emphasizes that post-trial communication is often the weakest part of the communication plan. As a result, deliberate communication activities are imperative to ensure that stakeholders conclude their trial experience positively, understand the results and the role they played in generating those results, and consider supporting/and or participating in clinical trials in the future.

## Conclusion

The aims of this project were to understand the current use of communication plans in clinical trials and to develop an easy-to-use communication plan template for investigators to incorporate alongside other trial documents such as protocols and SAPs. Our quantitative findings showed a clear gap in the inclusion of communication plans in clinical trials, with no single trial contributing a communication plan with the study protocol.

Qualitative interviews revealed enthusiasm for a communication plan template, and through interviews with clinical trial experts, we identified several key themes, including comments on community engagement, common barriers and challenges, and types of communication channels that are most effective when designing and delivering messages to clinical trial participants and the broader community. Further, experts interviewed emphasized the connection between accessible and inclusive participant communication and the research team’s ability to build trust with the community, paving the way for future research trials and engagement.

### Strengths and limitations

To our knowledge, this is the first in-depth analysis of communication plans in clinical trials to date. We conducted rigorous data mining of publicly available clinical trial protocols fitting our inclusion and exclusion criteria. This data, along with material from informative interviews with clinical trial experts, provided us with rich information and valuable suggestions for the development of the communication plan template. One limitation of this study was the lack of involvement of clinical trial participants in the design of the communication plan template. In addition, a brief survey was sent to interview participants to obtain data on their knowledge and perceptions of benefit sharing, but only one participant responded. Our sample size of publicly available clinical trial protocols was also limited. As this was the first version of the communication plan template, and because it outlines a set of minimum acceptable components, we advocate for its use and feedback for future iterations. Utilizing the communication plan template should be considered a best practice in clinical trials.

## Supplementary Information

**Additional file 1.** Interview guide for clinical trial experts

## Data Availability

Protocols used to mine for communication language are available here: https://dac-trials.tghn.org/resources/protocol-library/

## Abbreviations

CAB: Community advisory board
FDAAA: Food and Drug Administration Amendments Act
HIV: Human immunodeficiency virus
LMIC: Low-and-middle-income countries
NIH: National Institutes of Health
SAP: Statistical analysis plan
TB: Tuberculosis
WHO: World Health Organization
