# Supplementary material for: Creating a best practice template for participant communication plans in global health clinical studies
Source: Trials. 2023 Mar 2;24:158. doi: 10.1186/s13063-023-07185-4 (PMC9983505; doi:10.1186/s13063-023-07185-4)
Supplement: Supplementary file 1 — Additional file 1. Interview guide for clinical trial experts [file 13063_2023_7185_MOESM1_ESM.docx]

**Appendix A. Interview Guide for Clinical Trial Experts.**

1. How many clinical trials have you been involved in? In what countries? What was your role?
2. Tell us a bit about how you were involved in any communication as part of the trial. This could be with any audience, trial participants, participant families/supporters, the community, or other stakeholders (community health, state/ region/ country health boards, etc.).?
3. From your perspective, are there any benefits of communication with trial participants throughout or after the study has been completed? With participant families/supporters? With the community? *(If no, skip to 8)*
4. What strategies have you used/seen to improve communication with clinical trial participants? in pre-trial comms? In during-trial comms? In post-trial comms? (with all cohorts) Are they successful?
   1. What, if any, have been typical channels used to communicate with trial participants? With participants’ families/supporters? With communities?
      *(subset or prompt for #5 to explore channels) Ask only if needed*
5. What key considerations are used to determine the best channels for communication? (established guidelines/ best practices? review of the literature?)
6. What do you think could be the biggest barriers to effective communication to clinical trial participants throughout the duration of a study?

Channels of communications different b/t women, vs. adolescents, men etc.

Adaptable throughout - re-evaluate whether the communications are working.

1. If you have not seen successful strategies for communication during trials, what would you recommend as best practices/minimum standards in communicating with participants pre-trial, during and post-trial? (Same for families/supporters and communities)

*For Facilitator Use Only: Probes for pre-trial communications: Channels like pre-trial public meetings, meetings with community elders/leaders/chiefs, door-to-door campaigns, languages used, minimum timeframes (ahead of trial)*

*Probes for during trial communications: Community advisory boards, community liaison officers, suggestion boxes, WhatsApp groups, text chains, languages, interpreters, social media*

*Probes for post-trial communications: Sharing results, timeframe, language, level of synthesis, where shared (pharma websites and registries seem to be the norm)*
